# Pioglitazone treatment mitigates cardiovascular bioprosthetic degeneration in a chronic kidney disease model

**DOI:** 10.3389/fphar.2024.1412169

**Published:** 2024-08-08

**Authors:** Shintaro Katahira, Mareike Barth, Robin Döpp, Yukiharu Sugimura, Vera Schmidt, Jessica Isabel Selig, Yoshikatsu Saiki, Joachim Jankowski, Nikolaus Marx, Willi Jahnen-Dechent, Artur Lichtenberg, Payam Akhyari

**Affiliations:** ^1^ Department of Cardiovascular Surgery, Medical Faculty, University Hospital Düsseldorf, Heinrich Heine University Düsseldorf, Dusseldorf, Germany; ^2^ Division of Cardiovascular Surgery, Tohoku University Graduate School of Medicine, Sendai, Japan; ^3^ Department of Cardiac Surgery, Medical Faculty, University Hospital RWTH Aachen, Aachen, Germany; ^4^ Department of Thoracic and Cardiovascular Surgery, West German Heart and Vascular Center, University of Duisburg-Essen, Essen, Germany; ^5^ Institute of Molecular Cardiovascular Research, Medical Faculty, RWTH Aachen University, Aachen, Germany; ^6^ Department of Internal Medicine I (Cardiology), University Hospital RWTH Aachen University, Aachen, Germany; ^7^ Helmholtz-Institute for Biomedical Engineering, University Hospital RWTH Aachen, Aachen, Germany

**Keywords:** chronic kidney disease (CKD), PPAR gamma agonist, pioglitazone, bioprosthetic graft, structural valve deterioration, bioprosthetic valve dysfunction, calcification, intima hyperplasia

## Abstract

**Aims:**

Chronic kidney disease (CKD) is a risk factor for the development of cardiovascular diseases, e.g., atherosclerosis and calcific aortic valve disease, leading inevitably to valve replacement surgery. CKD patients with bioprosthetic cardiovascular grafts, in turn, have a higher risk of premature graft degeneration. Peroxisome proliferator-activated receptor gamma (PPARγ) activation by pioglitazone has cardio-renal protective properties, and research using a heterotopic valve implantation model has shown anti-degenerative effects of PPARγ activation on bioprosthetic valved grafts (BVG) in rats. The present work aims to analyze a potential protective effect of pioglitazone treatment on BVG in an adenine-induced rat model of CKD.

**Methods and Results:**

BVG of Sprague Dawley rats were heterotopically implanted in Wistar rats in an infrarenal position for 4 and 8 weeks. Animals were distributed into three groups for each time point: 1) control group receiving standard chow, 2) CKD group receiving 0.25% adenine and 3) CKD + pioglitazone group (300 mg per kg of 0.25% adenine chow). BVG function was analyzed by echocardiography. Plasma analytes were determined and explanted grafts were analyzed by semi-quantitative real-time PCR, Western blot analysis, histology and immunohistology.

PPARγ activation significantly reduced CKD-induced calcification of aortic and valvular segments of BVG by 44% and 53%, respectively. Pioglitazone treatment significantly also reduced CKD-induced intima hyperplasia by 60%. Plasma analysis revealed significantly attenuated potassium and phosphate levels after pioglitazone treatment. Moreover, PPARγ activation led to significantly decreased interleukin-6 gene expression (by 57%) in BVG compared to CKD animals. Pioglitazone treatment leads to functional improvement of BVG.

**Conclusion:**

This study broadens the understanding of the potential value of PPARγ activation in cardio-renal diseases and delineates pioglitazone treatment as a valuable option to prevent bioprosthetic graft failure in CKD. Further mechanistic studies, e.g., using small molecules activating PPARγ signaling pathways, are necessary for the evaluation of involved mechanisms. Additionally, the translation into pre-clinical studies using large animals is intended as the next research project.

## 1 Introduction

Due to an increase in the incidence and prevalence of cardiovascular risk factors like diabetes (Sun et al., 2022) and hypertension (Fuchs and Whelton, 2020), the number of patients with chronic kidney disease (CKD) is growing constantly, which, in turn, represents a burden to healthcare systems globally (GBD 2017 Disease and Injury Incidence and Prevalence Collaborators, 2018; Marassi and Fadini, 2023). Patients with CKD in turn are at higher risk of developing cardiovascular diseases (CVD) with atherosclerotic and calcific lesions of the arteries and the aortic valve [(Rong et al., 2018; Abd et al., 2015), reviewed in (Jankowski et al., 2021)]. The latter condition often necessitates surgical or interventional replacement of the aortic valve, which in CKD patients bears a high risk of post-operative complications (Rivera et al., 2023). Moreover, the choice between mechanical or bioprosthetic valve replacement requires substantial discussion and individual decisions (Ju et al., 2017; Kim et al., 2022). CKD patients with bioprosthetic heart valve grafts, however, have an increased risk of premature structural valve deterioration (SVD) and finally bioprosthetic valve failure (Ternacle et al., 2019; Garcia et al., 2021; Genereux et al., 2021).

Pioglitazone is a “peroxisome proliferator-activated receptor γ” (PPARγ) agonist, which is clinically used as an insulin-sensitizer in type 2 diabetes (Yki-Järvinen, 2004). Despite its hypoglycemic properties, pioglitazone features also protective effects in CVD (Bell and Jerkins, 2023) as well as in CKD (Sun et al., 2017; Papaetis, 2022). In detail, pioglitazone decreases inflammation (Hong et al., 2010; Park et al., 2011; Zou et al., 2021; Lee et al., 2022), ameliorates degenerative processes like neointimal proliferation (Takagi et al., 2009; Hong et al., 2010), as well as arterial and valvular calcification (Li et al., 2012; Chu et al., 2013; Zhu et al., 2021) by attenuating the atherosclerotic burden (Nissen et al., 2008; Yamamoto et al., 2015). Own studies using a heterotopic valve implantation model in rats have shown that pioglitazone is able to alleviate degenerative processes in terms of inflammation and calcification in bioprosthetic valved grafts (BVG) (Assmann et al., 2021; Katahira et al., 2021; Assmann A. K. et al., 2022). Furthermore, this protective anti-degenerative effect of PPARγ activation has been demonstrated to persist in front of pro-degenerative co-morbidities like metabolic syndrome (Assmann A. K. et al., 2022) and diabetes (Katahira et al., 2021).

Hence, pioglitazone is a promising candidate in the prevention of SVD and, thus bioprosthetic valve failure in CKD patients. In the present work, we used the previously well-characterized model to examine potential anti-degenerative effects of PPARγ activation on BVG in a CKD model.

## 2 Methods

### 2.1 Animals and experimental design

Animal experiments were conducted in the central animal care facility of the University of Düsseldorf [“*Zentrale Einrichtung für Tierforschung und wiss*. *Tierschutzaufgaben*” (ZETT), Heinrich Heine University Düsseldorf, Germany] following institutional and state guidelines and in agreement with the national animal welfare act and approved by the local state animal care committee (Landesamt für Natur, Umwelt und Verbraucherschutz NRW; reference number G182/17). All procedures conformed to the guidelines from Directive 2010/63/EU of the European Parliament on the protection of animals used for scientific purposes.

**FIGURE 1:**
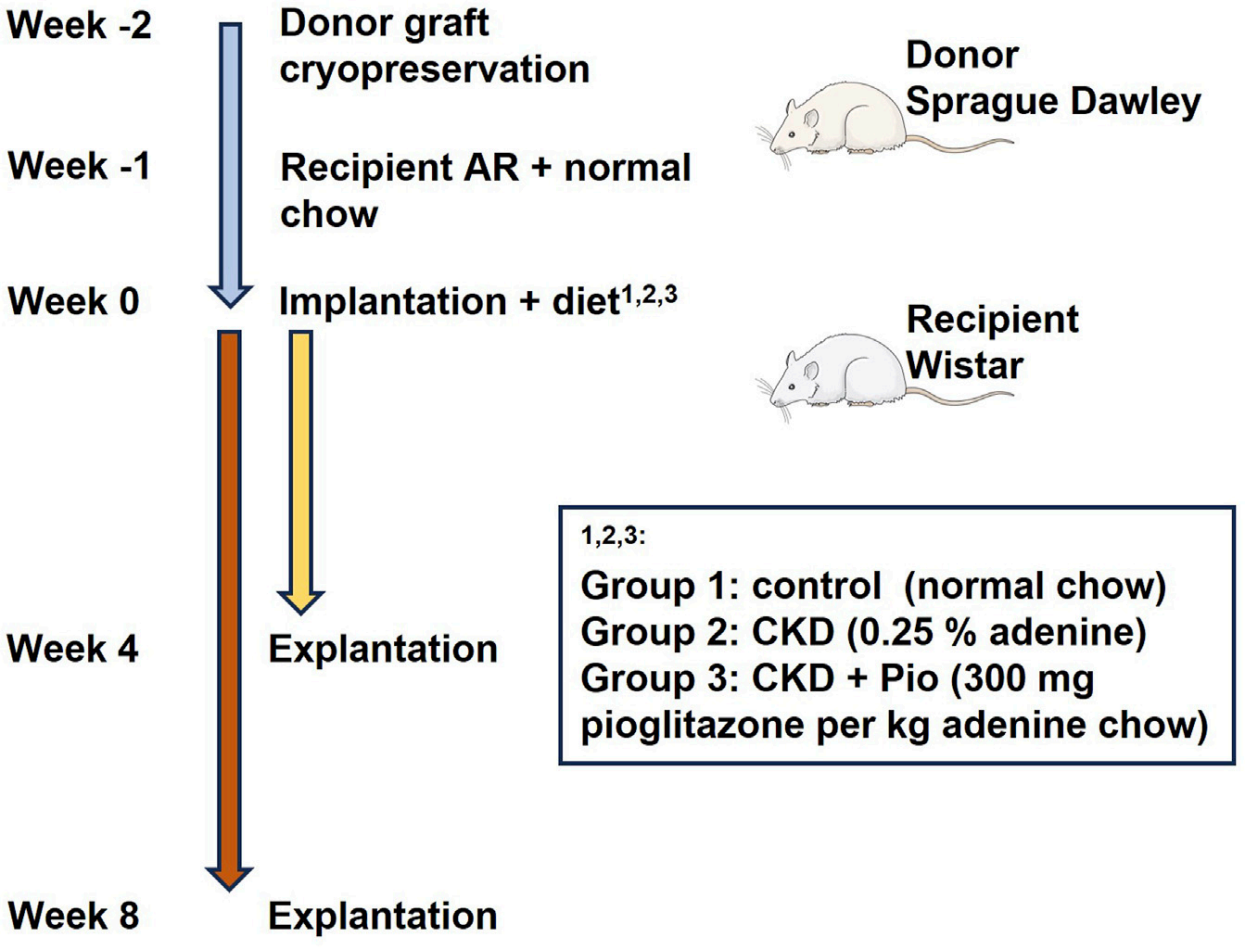
Schematic presentation of the workflow, timeline, and treatment groups. AR, Aortic regurgitation; CKD, chronic kidney disease; Pio, pioglitazone.

The experimental design is shown in Figure 1. Donor valve-bearing aortic conduits were prepared from male Sprague Dawley rats (n = 69). In detail, donor animals received 10 mg/kg xylazine hydrochloride and 100 mg/kg ketamine i.p. for general anesthesia. After sternotomy, the animals were euthanized by bleeding. Afterwards, the hearts were extracted, and the aortic conduits were dissected and stored at −80°C as previously described (Assmann et al., 2014a; Katahira et al., 2021) and as described to more detail in Section 2.2.

Recipient male Wistar rats (n = 69) underwent surgical induction of native aortic valve regurgitation (AR). In detail, recipient animals received general anesthesia by inhalation anesthesia with 2,0 – 2,5 vol% with isoflurane. Analgesia was performed intra-operatively by s.c. application of 5 mg/kg carprofen. Then, induction of native aortic valve regurgitation was performed as described before (Munakata et al., 2013; Assmann et al., 2014b; Katahira et al., 2021) and as described to more detail in Section 2.3. Post-operative analgesia was performed by s.c. application of 5 mg/kg carprofen and s.c. application of 5 mg/kg carprofen was performed until day 5 post-operatively every 24 h.

One week after induction of AR, the animals received heterotopic implantation of donor valve-bearing aortic conduits [now referred to as “bioprosthetic valved grafts” (BVG)]. Therefore, the animals received general anesthesia by inhalation anesthesia with 2,0 – 2,5 vol% with isoflurane. Analgesia was performed intra-operatively by s.c. application of 5 mg/kg carprofen. Surgical implantation of BVG was performed as described before (Munakata et al., 2013; Assmann et al., 2014b; Katahira et al., 2021) and as described to more detail in Section 2.4. Post-operative analgesia was performed by s.c. application of 5 mg/kg carprofen and s.c. application of 5 mg/kg carprofen was performed until day 5 post-operatively every 24 h.

Recipients were then randomly divided into three groups receiving different diets fed ad libitum: Rats fed with normal diet were appointed as control group (n = 20). Rats fed with 0.25% adenine to induce the CKD model were appointed as CKD group (n = 20). Rats fed with 0.25% adenine further supplemented with pioglitazone (300 mg per kg of diet chow) were appointed as CKD + Pio group (n = 20). Animals were sacrificed after 4 and 8 weeks (n = 10 at each time point and of each group) and BVG as well as blood samples were collected. Therefore, animals received general anesthesia by inhalation anesthesia with 2,0 – 2,5 vol% with isoflurane. After sternotomy, the animals were euthanized by bleeding. Afterwards, the BVG were dissected, prepared according to their further analysis, and stored at −80°C as described previously (Munakata et al., 2013; Assmann et al., 2014b; Katahira et al., 2021).

### 2.2 BVG preparation

BVG were prepared 1–2 weeks prior to implantation. For preparation of the grafts, Sprague Dawley donor rats (n = 69; 300–350 g) received general anesthesia by i.p. injection of 10 mg/kg xylazine hydrochloride and 100 mg/kg ketamine. After sternotomy, the animals were euthanized by bleeding, followed by thoracotomy and dissection of the valve-bearing aortic roots (see also Section 2.1). Therefore, the heart and the aortic arch were carefully removed, rinsed with PBS and ventricular myocardial tissue was resected under a microscope to prepare the BVG. The proximal segment of the left and right coronary arteries of the roots were ligated with 8-0 Prolene and the grafts were stored at −80°C in a pre-cooled conservation medium consisting of Dulbecco’s modified Eagle’s medium, 10% dimethyl sulfoxide and 20% fetal calf serum as previously described (Assmann et al., 2014a).

### 2.3 Induction of aortic valve regurgitation

To support a physiological opening and closure movement of the implanted BVG in a heterotopic position, a controlled induction of AR was performed as previously described (Munakata et al., 2013). Therefore, recipient Wistar rats (n = 69; 250–350 g) received a retrograde wire-induced perforation of the aortic valve by a catheter via the right carotid artery under inhalative anesthesia with 2 – 2.5 vol% isoflurane (see also Section 2.1) and constant echocardiographic guidance (Philips HDX11). The resulting moderate AR leads to reversal blood flow which then ensures proper leaflet closure of the implanted BVG (Légaré et al., 2000; Légaré and Ross, 2004; Munakata et al., 2013). During surgery, 1 mL blood was collected for further analysis.

### 2.4 Implantation of BVG

One week after induction of AR, Doppler-analysis of the native aortic valve and assessment of the severity of AR was performed in general anesthesia by inhalation anesthesia with 2,0 – 2,5 vol% with isoflurane (see Section 2.1), directly prior to implantation of BVG into the abdominal aorta as previously described (Munakata et al., 2013; Katahira et al., 2021). For implantation, the grafts were thawed at room temperature and rinsed in PBS three times prior to use. In brief, the graft was implanted to the abdominal aorta distal to the renal artery by anastomoses. Afterwards, the abdominal aorta was ligated between the distal and the proximal anastomoses to ensure total aortic blood flow through the BVG.

### 2.5 Analysis and group sizes

After 4 and 8 weeks, respectively, animals received general anesthesia by inhalation anesthesia with 2,0 – 2,5 vol% with isoflurane (see also Section 2.1) and echocardiography of the BVG was performed. Animals were sacrificed by bleeding (see also Section 2.1). Afterwards, grafts were explanted, kidneys and blood from the inferior vena cava were taken and processed for further analysis. The body weight as well as the weight of left and right kidneys was evaluated from n = 66 animals (i.e., n = 30 at 4 weeks and n = 36 at 8 weeks time point, resulting in n = 10–12 per subgroup). For standard clinical plasma parameters, plasma samples from n = 66 animals (i.e., n = 31 at 4 weeks and n = 35 at 8 weeks time point, resulting in n = 10–12 per subgroup) were analyzed. Adiponectin ELISA was performed using plasma from n = 69 animals (i.e., n = 33 at 4 weeks and n = 35 at 8 weeks time point, resulting in n = 11–12 per subgroup). IL-6, IL1-β and TNFα ELISA was performed using plasma from n = 65 animals (i.e., n = 30 at 4 weeks and n = 35 at 8 weeks time point, resulting in n = 10–12 per subgroup). Western blot analysis of fetuin-A protein levels was performed using plasma from n = 36 animals (i.e., n = 18 per time point, resulting in n = 6 per subgroup). For histology, explanted grafts from n = 24 animals (i.e., n = 12 per time point, resulting in n = 4 per subgroup) were used. For mRNA analysis explanted grafts of n = 38 animals (i.e., n = 19 per time point, resulting in n = 6-7 animals per subgroup) were used. Mean values were used for analysis.

#### 2.5.1 Histology

Explanted BVG appointed for histology were divided into four sections directly after explantation: Aortic root and aortic valve region (A1), ascending aorta to proximal aortic arch region (A2), distal aortic arch region (B1), and descending aorta region (B2). Each section was placed into cryomolds, embedded into cryo-compound, and shock-frozen in 2-methyl butane cooled to the temperature of liquid nitrogen. For histology and immunohistology, 5 μm tissue sections were prepared. Hematoxylin and eosin, alizarin and von Kossa staining was performed and analyzed as described in detail before (Katahira et al., 2021). Moreover, immunohistochemistry using the DAB Substrate Kit (Zytomed, Berlin, Germany) for the detection of RAGE expression was performed (anti-RAGE, cat. no.: ab3611; Abcam, Cambridge, United Kingdom and goat-anti-rabbit secondary antibody, cat. no.: 111.035.003; Jackson ImmunoResearch, Cambridgeshire, United Kingdom). Picrosirius red/fast green staining was performed according to conventional standard protocols using 5 µm tissue sections of left and right kidneys. Staining of Alizarin and von Kossa was documented and processed blinded using pseudonymized labelling of specimen slides. Micrographs were taken using a Leica DM2000 and Leica LAS software Version 3.8.0. Von Kossa staining score was performed as previously described (Assmann A. et al., 2022). Alizarin staining score was adapted to the von Kossa staining score described in detail before (Assmann et al., 2014b). Analysis and presentation were performed using as well as Fiji/ImageJ2 software and GraphPad Prism version 8.0 (GraphPadSoftware, San Diego, CA, United States).

#### 2.5.2 mRNA analysis

Changes in mRNA expression of whole grafts (A1-B2 regions) were evaluated as previously described (Katahira et al., 2021). In brief, BVG were explanted, shock-frozen in liquid nitrogen and stored at −80°C upon analysis. For analysis of mRNA expression, samples were homogenized, mRNA was isolated using TRIzol Reagent (Invitrogen, Carlsbad, United States) and mRNA quality was assessed by measuring mRNA integrity (Agilent RNA 6000 Nano Kit; Agilent Technologies, Santa Clara, United States). Reverse transcription of mRNA to cDNA was performed using QuantiTect Reverse Transcription Kit (Qiagen, Hilden, Germany) according to the manufacturer’s protocol. Semi-quantitative mRNA expression analysis was performed according to the ΔΔCt method using a StepOnePlus cycler (Applied Biosystems, Foster City, United States) and Platinum SYBR Green qPCR SuperMix-UDG/ROX kit (Invitrogen, Carlsbad, CA, United States) as previously described (Selig et al., 2019). Primers were obtained from Invitrogen (Carlsbad, United States; sequences listed in Table 1) and the mRNA expression of tumor necrosis factor alpha (TNFα), interleukin-1 beta (IL-1β), interleukin-6 (IL-6), receptor for advanced glycation end products (RAGE), tissue non-specific alkaline phosphatase (ALPL), osteopontin (OPN), osteocalcin (OCN), bone morphogenic protein 2 (BMP2), runt-related transcription factor 2 (RUNX2), and solute carrier family 20 member 1 (PIT-1) was analyzed. 60S ribosomal protein L13a (RPL13A), 18S ribosomal RNA (18S) and actin beta (ACTB) were used as housekeeping genes.

**TABLE 1:** Primer sequences.

Gene	Forward sequences (*5′-3′*)	Reverse sequences (*5′-3′*)	
TNFα	*GCT​CCC​TCT​CAT​CAG​TTC​CA*	*GCT​TGG​TGG​TTT​GCT​ACG​AC*	
IL-1β	*AGG​ACC​CAA​GCA​CCT​TCT​TT*	*CAT​CAT​CCC​ACG​AGT​CAC​AG*	
IL-6	*ACC​ACC​CAC​AAC​AGA​CCA​GT*	*AGT​GCA​TCA​TCG​CTG​TTC​AT*	
RAGE	*TGA​ACT​CAC​AGC​CAA​TGT​CC*	*TCAGAGGTTTCCAAG*	
ALPL	*GCA​CTC​CCA​CTA​TGT​CTG​GAA*	*AGG​GAA​GGG​TCA​GTC​AGG​TT*	
OPN	*AAG​CCT​GAC​CCA​TCT​CAG​AA*	*ATG​GCT​TTC​ATT​GGA​GTT​GC*	
OCN	*AAG​CAG​GAG​GGC​AGT​AAG​GT*	*GTC​CGC​TAG​CTC​GTC​ACA​AT*	
BMP2	*GCT​CAG​CTT​CCA​TCA​CGA​A*	*AAG​AAG​AAG​CGT​CGG​GAA​GT*	
RUNX2	*GAT​GAC​ACT​GCC​ACC​TCT​GA*	*GAT​GAA​ATG​CCT​GGG​AAC​TG*	
PIT-1	*TGT​ATT​GTC​GGT​GCA​ACC​AT*	*TGG​AGA​GAC​GAA​CCA​AGA​CA*	
RPL13A	*GAT​CCC​ACC​ACC​CTA​TGA​CA*	*CTT​CAG​ACG​CAC​AAC​CTT​GA*	
18S	*GCA​ATT​ATT​CCC​CAT​GAA​CG*	*GGC​CTC​ACT​AAA​CCA​TCC​AA*	
ACTB	*CCC​GCG​AGT​ACA​ACC​TTC​T*	*CGTCATCCATGGCGAACT*	

#### 2.5.3 Plasma analysis

At the time of AR as well as at the time of BVG explantation at 4 weeks and 8 weeks, respectively, blood samples were collected from the inferior vena cava using a heparin-containing syringe. Samples were centrifuged (4°C, 1,000 rpm, 15 min) and plasma was analyzed at the Institute of Clinical Chemistry and Laboratory Diagnostics, Medical Faculty, Heinrich Heine University, Düsseldorf, Germany. The following analytes have been measured: Sodium, calcium, phosphate, potassium, creatinine, urea, uric acid, total cholesterol, triglycerides, high-density lipoprotein (HDL), low-density lipoprotein (LDL), glucose, aspartate aminotransferase (AST) and alanine aminotransferase (ALT). For plasma analysis, see Table 2.

**TABLE 2:** Body/kidney weight and plasma parameter Plasma parameter measured at explantation time point at 4 and 8 weeks with values representing mean ± standard error of mean. Animals included: 4 weeks n = 11 in each group; 8 weeks n = 12 in each group.

4 weeks	Control	CKD	CKD + Pio	*p*-value CKD vs. CDK + Pio
Body weight (g)	413 ± 11	356 ± 8***	392 ± 6	0.0021
Kidney weight (g)	1.4 ± 0.04	2.5 ± 0.1***	2.5 ± 0.1***	ns
Crea (mg/dL)	0.2 ± 0.01	0.9 ± 0.1****	0.7 ± 0.03**	ns
Urea (mg/dL)	38.5 ± 1.6	21.2 ± 3.3***	27.4 ± 2.96*	ns
UA (mg/dL)	0.35 ± 0.03	0.42 ± 0.06	0.46 ± 0.05	ns
Na (mmol/L)	142 ± 1.5	145 ± 1.0	146 ± 1.1	ns
K (mmol/L)	3.6 ± 0.2	4.2 ± 0.1*	4.1 ± 0.1	ns
Ca (mmol/L)	2.2 ± 0.1	2.3 ± 0.1	2.4 ± 0.1	ns
P (mg/dL)	2.1 ± 0.2	2.8 ± 0.1**	2.5 ± 0.1	ns
TC (mg/dL))	56.9 ± 4.8	66.7 ± 2.7	73.6 ± 4.3*	ns
TG (mg/dL)	99.2 ± 11.9	90.7 ± 8.2	95.3 ± 8.8	ns
LDL (mg/mL)	12.3 ± 1.5	16.5 ± 1.7	19.7 ± 1.2**	ns
HDL (mg/dL)	33.6 ± 3.6	39.4 ± 2.2	45.0 ± 3.4	ns
Glucose (mg/dL)	283 ± 9	277 ± 14	256 ± 15	ns
AST (IU/L)	58.1 ± 2.7	59.3 ± 3.3	62.2 ± 3.6	ns
ALT (IU/L)	44.2 ± 2.6	29.9 ± 2.6**	30.9 ± 2.1*	ns

8 weeks	Control	CKD	CKD + Pio	*p*-value CKD vs. CDK + Pio
Body weight (g)	475 ± 11	359 ± 9****	381 ± 11**	ns
Kidney weight (g)	1.3 ± 0.03	2.4 ± 0.09****	2.3 ± 0.07***	ns
Crea (mg/dL)	0.3 ± 0.02	1.9 ± 0.1***	2.2 ± 0.2****	ns
Urea (mg/dL)	44.3 ± 1.9	45.0 ± 2.6	53.4 ± 5.3	ns
UA (mg/dL)	0.33 ± 0.02	0.48 ± 0.2	0.5 ± 0.1	ns
Na (mmol/L)	143 ± 0.6	144 ± 0.8	145 ± 1.4	ns
K (mmol/L)	3.4 ± 0.1	5.1 ± 0.4****	4.2 ± 0.1**	0.0446
Ca (mmol/L)	2.2 ± 0.1	2.1 ± 0.04	2.1 ± 0.1	ns
P (mg/dL)	1.8 ± 0.1	3.5 ± 0.2****	2.9 ± 0.1**	0.0304
TC (mg/dL))	56.8 ± 3.3	94.8 ± 5.8**	101.4 ± 4.1****	ns
TG (mg/dL)	65.1 ± 7.8	100 ± 15.5	96.9 ± 7.7*	ns
LDL (mg/mL)	13.3 ± 0.7	26.8 ± 1.8**	34.8 ± 1.9****	0.0042
HDL (mg/dL)	31.3 ± 3.1	55.7 ± 3.01***	57.8 ± 3.3***	ns
Glucose (mg/dL)	284 ± 9	225 ± 10*	196 ± 9****	0.0455
AST (IU/L)	62.6 ± 3.6	62.6 ± 8.9	63.8 ± 5.1	ns
ALT (IU/L)	39.9 ± 3.3	36.6 ± 3.1	28.6 ± 1.3*	0.0487

Crea, creatinine; UA, uric acid; Na, sodium; K, potassium; Ca, calcium; P, phosphate; TC, total cholesterol; TG, triglycerides; LDL, low density lipoprotein; HDL, high density lipoprotein; AST, aspartate aminotransferase; ALT, alanine aminotransferase; CKD, chronic kidney disease; Pio, pioglitazone; *: *p* < 0.05; **: *p* < 0.01; ***: *p* < 0.001; ****: *p* < 0.0001 (Control vs. CKD or CKD + Pio); *p*-values delineate pairwise comparison of CKD and CKD + Pio group using Mann-Whitney U test.

Levels of circulating fetuin-A were determined by reducing SDS-PAGE (10% acrylamide) and immunoblotting of 0.1 µL plasma per lane and a low molecular weight calibration kit (Cytiva; cat. no.: 17-0446-01). Western blot analysis was performed using K45 rabbit antiserum raised against recombinant rat fetuin-A (Denecke et al., 2003), secondary antibody (Dako; cat. no.: P0217) and enhanced chemiluminescence ECL reagent. Chemiluminescence was detected using a Fuji LAS-4000 scanner and quantification of scans was performed against identical rat serum samples run in duplicates on all gels as a loading control. ImageJ and GraphPad Prism software packages were used for signal quantification and statistical analysis of the scans, respectively.

#### 2.5.4 Colorimetric sandwich ELISA

For detection of IL-6, IL1-β and TNFα in plasma, Rat IL-6 Quantikine ELISA Kit (R&D Systems; cat. no.: R6000B), Rat IL-1 beta/IL-1F2 Quantikine ELISA Kit (R&D Systems; cat. no.: RLB00) and Rat TNF-alpha Quantikine ELISA Kit (R&D Systems; cat. no.: RTA00) were used according to the manufacturer’s instructions. Successful PPARγ activation by pioglitazone was performed by analysis of adiponectin Rat Total Adiponectin/Acrp30 Quantikine ELISA Kit (R&D Systems; cat. no.: RRP300) which has been shown to be a direct transcriptional target of PPARγ activation (Maeda et al., 2001) mediated by a PPAR-responsive element in the adiponectin promotor (Iwaki et al., 2003).

#### 2.5.5 Echocardiographic analysis

For evaluation of severity of native aortic valve insufficiency, the ratio of time-velocity integral of reversed diastolic flow (RVTI) to forward systolic flow (VTI) in the ascending aorta was measured as described before (Munakata et al., 2013; Assmann A. K. et al., 2022). Functional degradation of the BVG was analyzed by measuring RVTI and VTI distally of the implanted BVG in the abdominal aorta (“postBVG”) and by calculation of the according RVTI/VTI ratio in the 8 weeks time point groups. Ratios of 0.5 – 0.7 were considered as AR grade II–III.

#### 2.5.6 Data presentation and statistical analysis

Graphs and statistics were performed using GraphPad Prism version 8.0. Three or more groups were compared using Kruskal-Wallis test with Dunn’s post-hoc test or Two-way ANOVA with Tukey’s post-hoc test. Pairwise comparisons were performed using Mann-Whitney U test. *P*-value < 0.05 were considered as statistically significant. Data is described as mean ± standard error of mean.

## 3 Results

### 3.1 Metabolic effects of CKD and pioglitazone treatment

Metabolic effects of CKD and pioglitazone were analyzed by evaluation of body and kidney weight, and plasma analytes (Table 2). Animals in the CKD group showed significant weight loss together with significantly increased kidney weight. Gross morphological inspection showed enlarged kidneys with a pale appearance indicating fibrotic degeneration in the CKD groups with an attenuated manifestation in the CKD + Pio group. Staining of cross-sections of the kidneys showed massive fibrotic changes by collagen alterations in both CKD groups with a less pronounced finding in the CKD + Pio group (Supplementary Figure S1). Measurements of changes in plasma showed that the applied adenine diet induced a robust CKD model with significantly increased creatinine, potassium, and phosphorous levels. Moreover, decreased glucose levels and elevated plasma lipids reflect common CKD complications.

Pioglitazone treatment then led to a significant decrease of potassium and phosphate levels as well as to a further reduction of glucose plasma levels. Interestingly, ALT levels were significantly reduced by pioglitazone treatment. Plasma lipids remained unchanged except for LDL levels which were significantly increased under PPARγ activation.

Adiponectin levels in plasma showed a significant increase by pioglitazone treatment in both time points when comparing CKD and CKD + Pio groups, indicative for successful PPARγ target gene activation (Maeda et al., 2001; Iwaki et al., 2003). CKD groups present with significantly increased adiponectin levels compared to control groups (Supplementary Figure S1).

Analysis of plasma fetuin-A by immunoblotting showed similar protein levels at all time points and in all experimental groups with a decreased fetuin-A level after 8 weeks in the CKD + Pio group compared to the corresponding control group (Supplementary Figure S1).

### 3.2 Calcification of the tubular aortic wall segments of the BVG

**FIGURE 2:**
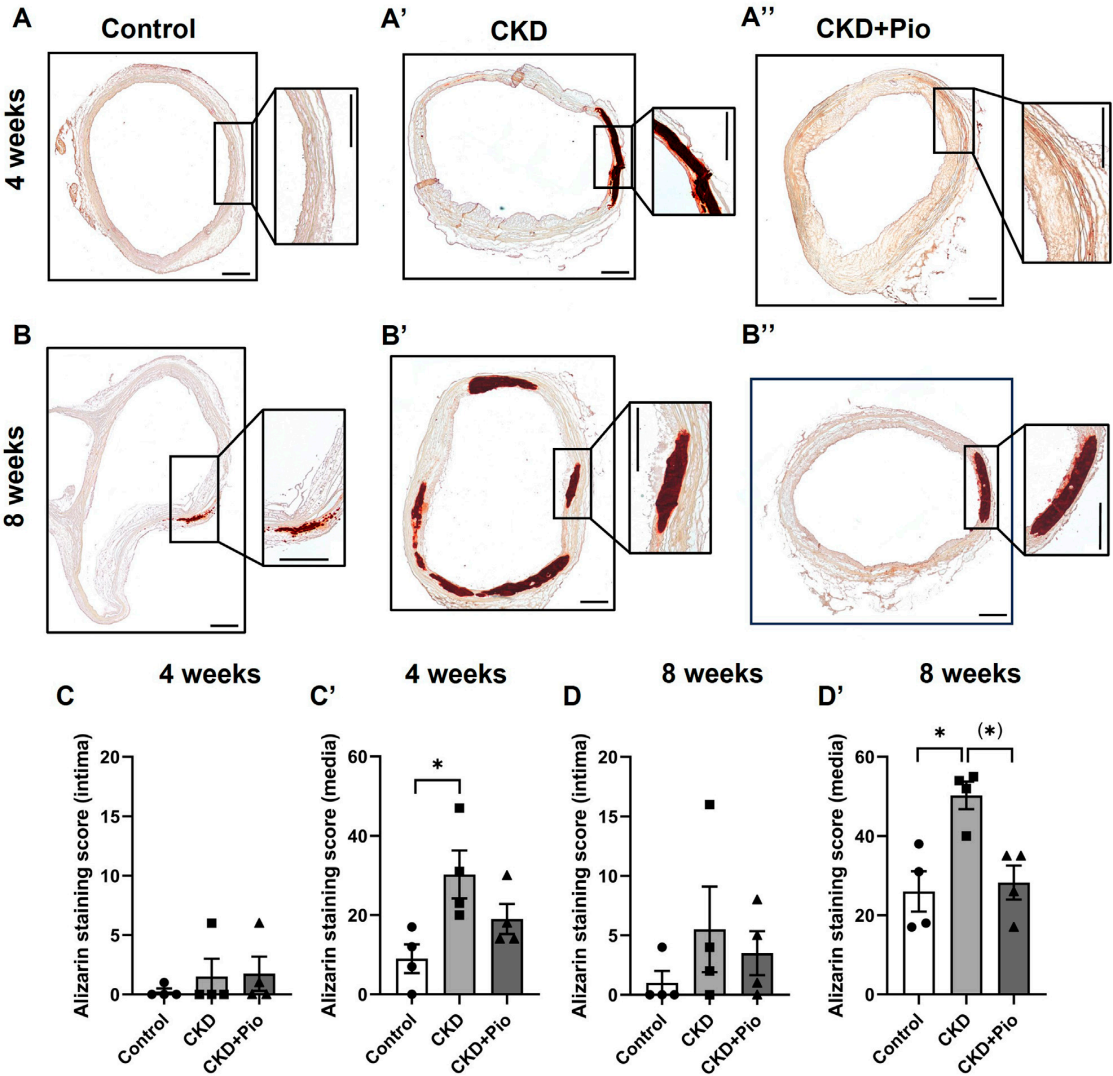
Alizarin staining of the aortic wall. Alizarin staining **(A–B”)** and quantification by Alizarin staining score **(C–D′)** of regions A2-B2 of the grafts. CKD leads to significantly accelerated calcifications in the media **(A′–D′)**. Pioglitazone treatment mitigates CKD-induced calcification after 8 weeks **(B, D′)**. Animals included: n = 4 in each subgroup with three sections of each sample. CKD, chronic kidney disease; Pio, pioglitazone; *: *p* < 0.05 using Kruskal-Wallis with Dunn’s post hoc test; (): pairwise comparison using Mann-Whitney U test. Bars: 400 µm.

To analyze potential spatial differences in calcific degeneration of the aortic wall, we analyzed calcium deposition in intima and media regions of tubular segments A2-B2 by Alizarin staining (Figure 2). Analysis of alizarin staining scores revealed generally low levels of positive staining as well high standard deviations in the individual segments (Supplementary Figure S2). Thus, in total intima segments (A2-B2) there were no differences in calcification levels, both at 4 weeks as well as at 8 weeks time points.

The media segments in total, however, showed significantly increased alizarin staining in the CKD group compared to control conditions at both time points. Pioglitazone treatment led to decreased calcium deposition at 4 weeks, although not statistically significant due to high variations in individual segments of the CKD group (Supplementary Figure S2). The protective effect of pioglitazone against calcification was more pronounced at 8 weeks, where pioglitazone treatment mitigates calcium deposition in CKD rats approximately to control levels.

Von Kossa staining and semiquantitative analysis of calcification thereupon showed comparable results (Supplementary Figure S3) although the statistical difference proved to be less clear due to a high variance in the individual segments (Supplementary Figure S4).

Besides calcification, the amount of intima hyperplasia was determined (Supplementary Figure S5). Quantification of intima thickness after 8 weeks revealed significant intima hyperplasia in the CKD group which was mitigated by pioglitazone treatment to the level of the according control group. Intima/media ratio remained unchanged after 4 weeks but was significantly higher in the CKD group at 8 weeks. Pioglitazone treatment led to a total reversal of intima/media increase in CKD rats over 8 weeks.

### 3.3 Calcification of the aortic valve of the BVG

**FIGURE 3:**
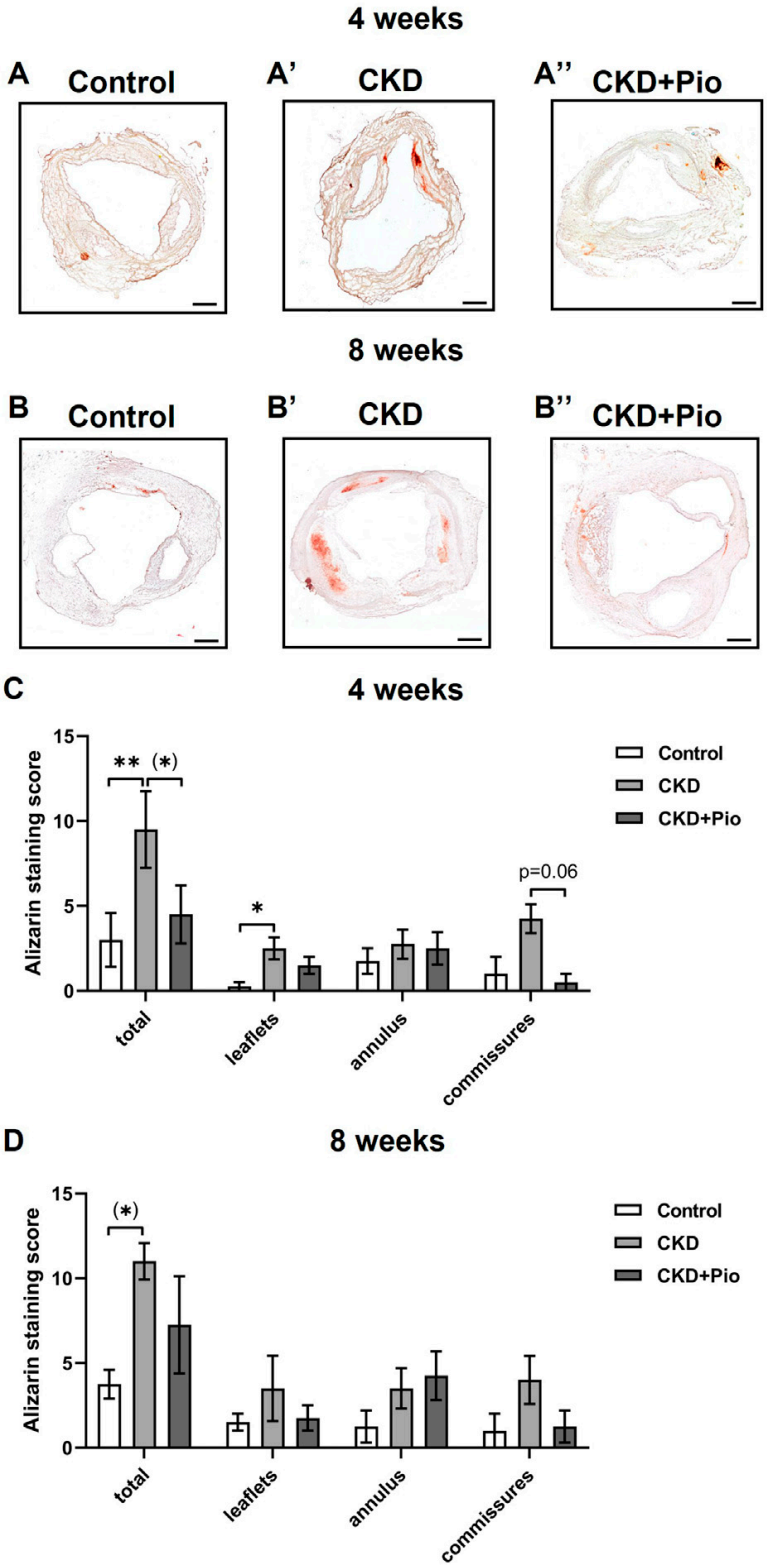
Alizarin staining of the bioprosthetic valve Alizarin staining **(A–B′)** and quantification by Alizarin staining score **(C, D)** of individual regions of the bioprosthetic valve. CKD partly leads to increased calcifications of the valve and pioglitazone treatment by trend mitigates CKD-induced calcification (in commissures: CKD vs. CKD + Pio; *p* = 0.06). Animals included: n = 4 in each subgroup with three sections of each sample. CKD, chronic kidney disease; Pio, pioglitazone; *: *p* < 0.05; **: *p* < 0.01 using Kruskal-Wallis with Dunn’s post hoc test; (): pairwise comparison using Mann-Whitney U test. Bars: 400 µm.

Analysis of Alizarin staining of the valvular part of the BVG revealed that CKD leads to increased calcium deposition while pioglitazone treatment leads to an attenuation of this effect (Figure 3). Differences between the treatment groups are most obvious in the 4 weeks time point when analyzing all three regions cumulatively (leaflets, commissures, and annulus) with significantly higher calcium deposition in the CKD group as compared to control and a by trend lower calcium deposition in the CKD + Pio group as compared to the CKD group (Figure 3C). Differences within the individual regions here are mainly present in the leaflet and commissure region of the bioprosthetic valve (see also corresponding von Kossa staining in Supplementary Figure S6). Due to higher dispersion of measured values in the individual parts of the valve, this observation is much less pronounced at the 8 weeks time point.

**FIGURE 4:**
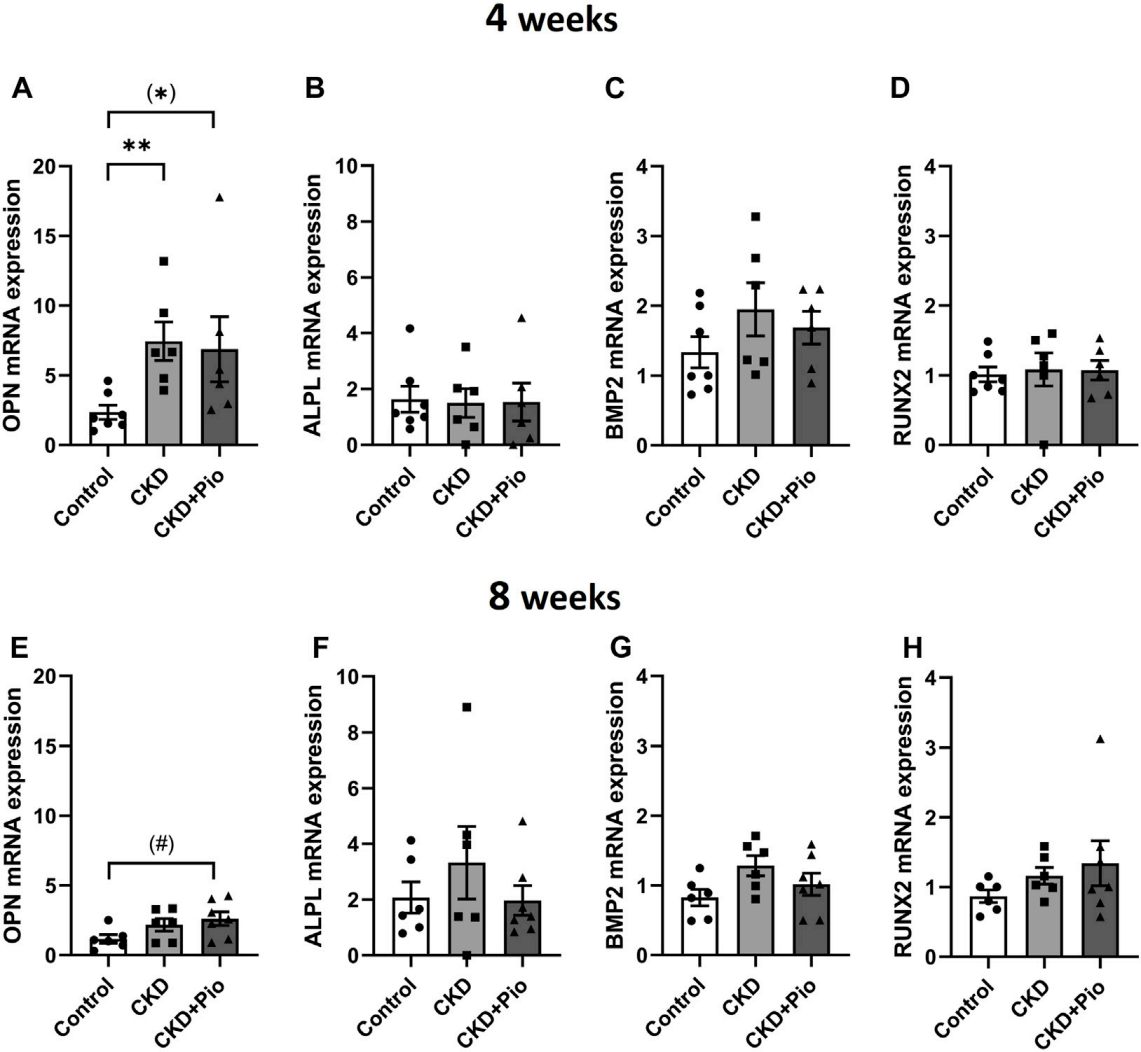
Chondro-osteogenic differentiation Gene expression of OPN **(A, E)**, ALPL **(B, F)**, BMP2 **(C, G)** and RUNX **(D, H)** in bioprosthetic grafts after 4 and 8 weeks. OPN shows significant upregulation by CKD and CKD + Pio treatment at 4 weeks time point **(A)**. ALPL, BMP2, and RUNX2 **(B–D,F–H)** show no altered gene expression. Animals included: 4 weeks control n = 7, CKD n = 6, CKD + Pio n = 6; 8 weeks control n = 6, CKD n = 6, CKD + Pio n = 7. CKD, chronic kidney disease; Pio, pioglitazone; OPN, osteopontin; ALPL, alkaline phosphatase; BMP2, bone morphogenic protein 2; RUNX2, runt-related transcription factor 2; *: *p* < 0.05; **: *p* < 0.01 using Kruskal-Wallis with Dunn’s post hoc test; #: *p* = 0.051; (): pairwise comparison using Mann-Whitney U test.

To investigate whether the protective effect of PPARγ activation is related to a downregulation of chondro-osteogenic differentiation markers of the BVG, whole grafts were analyzed by semi-quantitative PCR (Figure 4). OPN was significantly upregulated only at the 4 weeks time point in the CKD group, without showing differential expression by pioglitazone treatment. Gene expression of ALPL, BMP2 and RUNX2 remained unchanged at both examined time points. Expression of the sodium-phosphate symporter PIT1 remained unchanged (not shown).

### 3.4 Inflammatory effects of CKD and pioglitazone treatment

**FIGURE 5:**
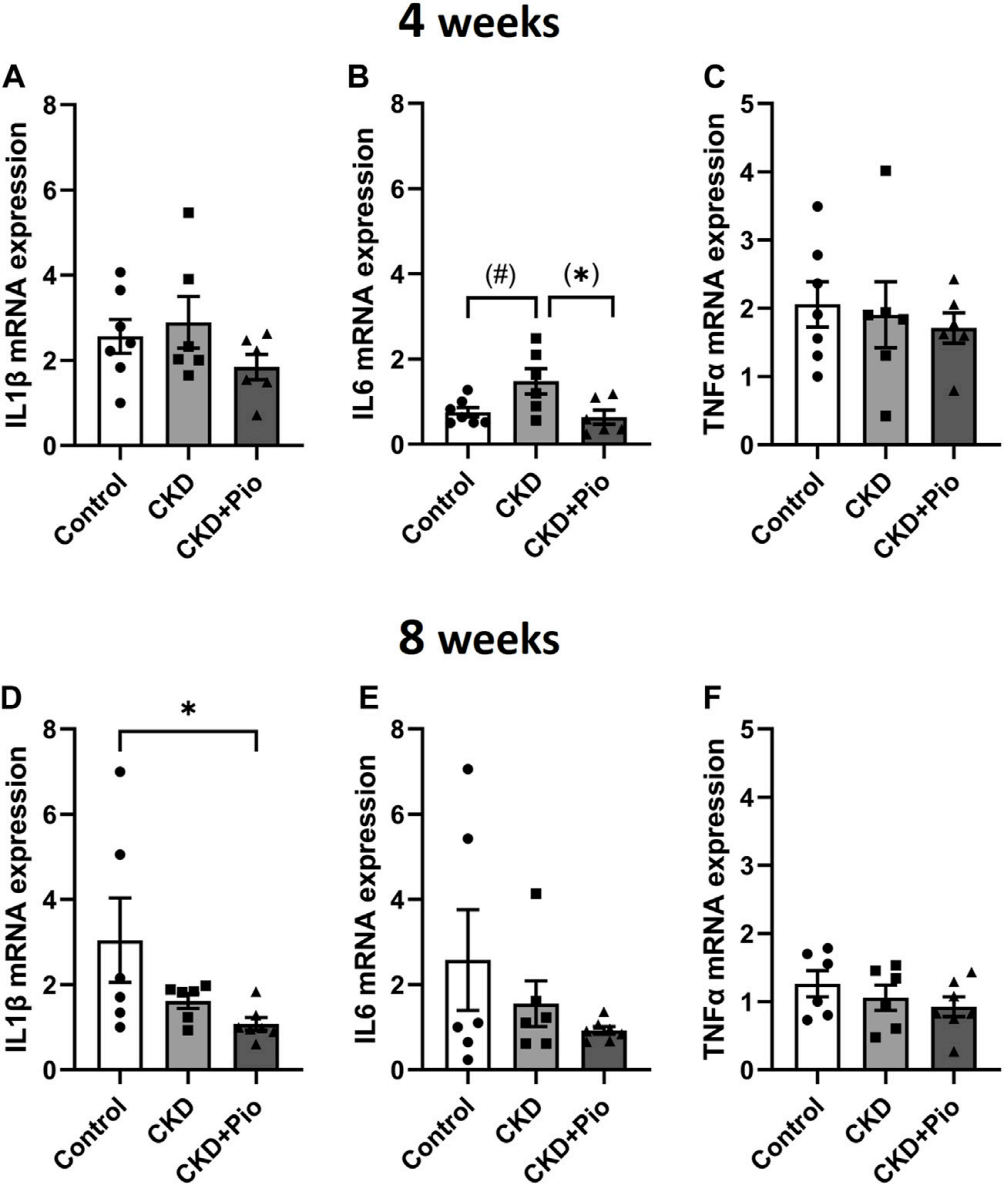
Expression of inflammatory markers Gene expression analysis of inflammatory markers at explantation time point at 4 weeks **(A–C)** and 8 weeks **(D–F)** with values being mean ± standard error of mean. IL6 expression is altered at 4 weeks and RAGE expression is altered at 8 weeks, IL1β and TNFα remain unchanged. Animals included: 4 weeks control n = 7, CKD n = 6, CKD + Pio n = 6; 8 weeks control n = 6, CKD n = 6, CKD + Pio n = 7. CKD, chronic kidney disease; Pio, pioglitazone; *: *p* < 0.05 using Kruskal-Wallis with Dunn’s post hoc test; #: *p* = 0.051; (): pairwise comparison using Mann-Whitney U test.

Inflammatory effects of CKD and pioglitazone treatment were evaluated by analysis of expression levels of cytokines IL1β, IL6 and TNFα in the graft material and in plasma. Whole BVG (A1-B2 region) were analyzed by semi-quantitative PCR and immunohistology. Here, the expression of certain markers revealed a time-dependent pattern (Figure 5). At 4 weeks, IL1β expression showed no significant differences between the treatment groups, while at 8 weeks, grafts of the CKD + Pio group showed a significantly decreased expression of IL1β in comparison to the control group. IL6 expression at 4 weeks showed a near significant increase in the CKD group compared to control and a significant decrease in the CKD + Pio group compared to the CKD group. TNFα expression remained unchanged both at 4 weeks as well as at 8 weeks.

Analysis of circulating cytokines in plasma revealed generally low levels of IL-6, IL-1β and TNFα levels beyond detection range (not shown).

Expression levels of RAGE, however, were unchanged at the early time point, but showed a generally higher expression at the 8 weeks time point (Supplementary Figure S7). Here, both the CKD as well as the CKD + Pio group showed higher gene expression levels by trend compared to the control group.

### 3.5 Functional evaluation of BVG under pioglitazone treatment

**FIGURE 6:**
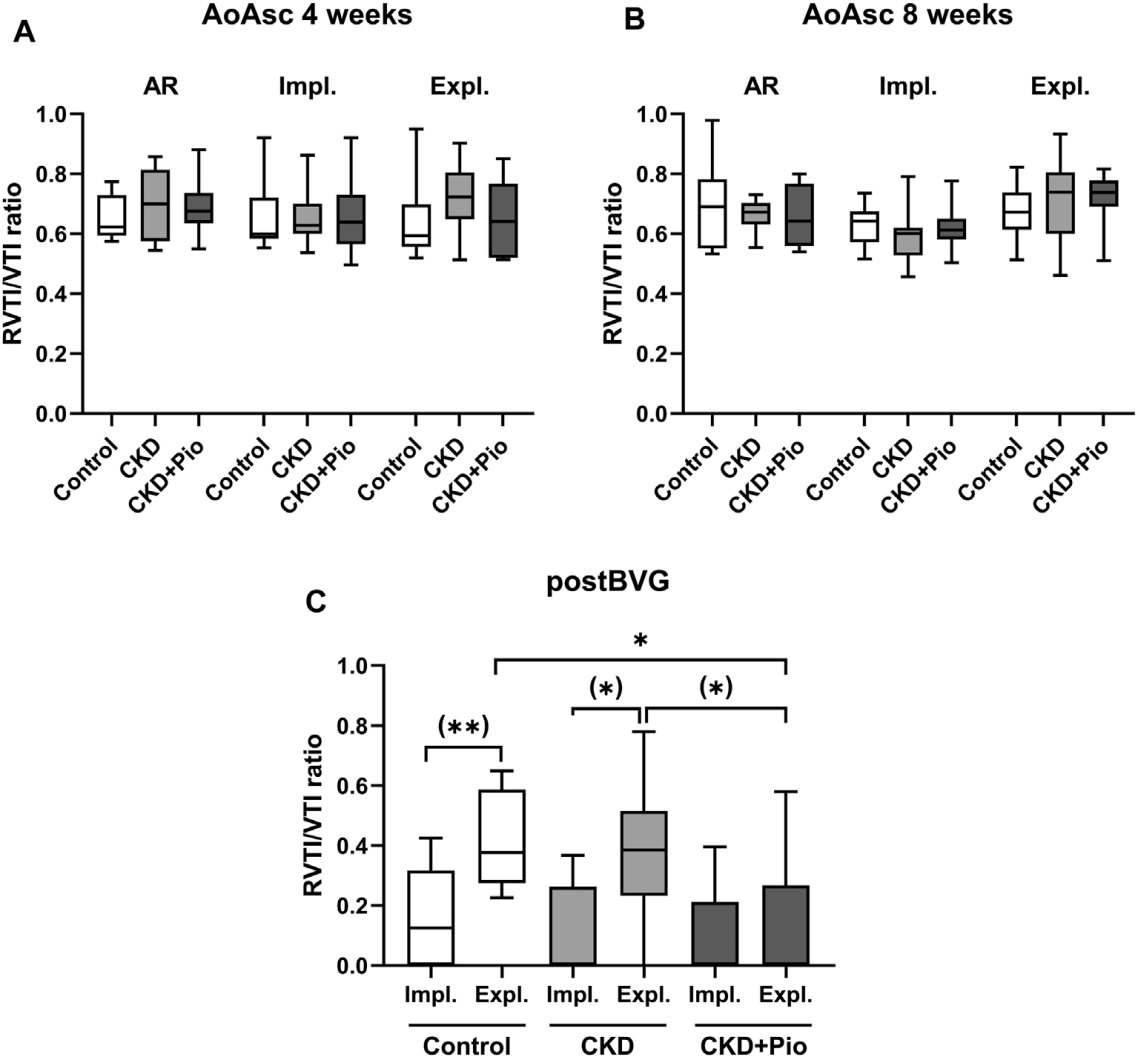
Echocardiographic analysis Echocardiographic data of RVTI/VTI ratios measured in the ascending aorta at 4 weeks **(A)** and 8 weeks **(B)** at time points of AR, implantation and explantation showing successful AR throughout treatment and duration. **(C)** RVTI/VTI ratios of implanted BVG directly after implantation and at the time point of explantation show significant AR in control and CKD group after 8 weeks with significantly improved function of BVG by pioglitazone treatment. RVTI, time-velocity integral of reversed diastolic flow; VTI, forward systolic flow; AoAsc, ascending aorta; AR, aortic valve regurgitation; Impl., implantation; Expl., explantation; postBVG, abdominal aorta distally of the implanted BVG; CKD, chronic kidney disease; Pio, pioglitazone; *: *p* < 0.05 using Kruskal-Wallis with Dunn’s post hoc test; (): pairwise comparison using Mann-Whitney U test.

Echocardiographic analysis shows successful induction of AR of the native aortic valve in both time points throughout AI induction, BVG implantation and explantation when measuring retrograde flow in the ascending aorta (Figures 6A+B).

RVTI/VTI ratios measured distally of the BVG of the 8 weeks groups show that the BVG at the time point of implantation is free from AR indicating an unimpaired blood flow (Figure 6C). At the time point of explantation, the control as well as the CKD group show significantly higher RVTI/VTI ratios when compared to the time point of implantation, whereas the CKD + Pio group presents no differences. The pioglitazone treated group exhibits significantly less AR at the time point of explantation compared to control and CKD group (Figure 6C), indicating a functional improvement of BVG by PPARγ activation.

## 4 Discussion

CKD is an important driver of SVD not only of the native valve but also of cardiovascular grafts and is in focus of clinical trials aiming at drug therapy (Barayev et al., 2023) and valve replacement interventions (Jacquemyn et al., 2023) without satisfying results.

In the present work we have used an adenine-induced CKD model in rats to study the impact of PPARγ activation on degenerative processes in BVG. For the first time, the results of this study show cardio-renal protective effects of pioglitazone administration on bioprosthetic cardiovascular implants in a model of CKD. With increasing preference of biological heart valves over mechanical prostheses for patients needing heart valve replacement, and due to significantly reduced durability of biological prostheses in patients with CKD, pioglitazone-induced anti-degenerative effects are of potential interest for the improvement of clinical outcomes in patients undergoing heart valve replacement.

### 4.1 Metabolic effects of pioglitazone treatment in the CKD model

Enhanced fluid retention in terms of increased plasma creatinine and sodium levels as a negative factor in chronic kidney disease due to pioglitazone (Satirapoj et al., 2018) was not observed. High potassium levels often occur in CKD patients (Collins et al., 2017; Costa et al., 2023) and have been described in adenine-induced CKD in animal models (Ashour et al., 2023). Interestingly, pioglitazone treatment led to ameliorative effects in our animals which might speak for a not yet described new renal-/cardio-protective role of pioglitazone since elevated potassium levels are associated with a higher risk for cardiovascular pathologies and mortality (Collins et al., 2017; Costa et al., 2023). Additionally, phosphate levels in plasma were upregulated in CDK groups, most prominently after 8 weeks. Hyperphosphatemia is a frequent complication in late-stage CKD and might worsen cardiovascular diseases (Barreto et al., 2019). In our model, pioglitazone treatment ameliorated this effect, which, to our knowledge, has not yet been reported. Calcium levels remained unchanged in all groups and time points, which is in accordance with other studies of adenine-induced CKD, even in high dose models [reviewed in (Shobeiri et al., 2010)]. Pioglitazone treatment led to a significant reduction in ALT levels compared to control and CKD. Alterations in liver enzymes like ALT due to CKD have been reported before (Ray et al., 2015) and pioglitazone has been reported to lower ALT levels in patients with non-alcoholic fatty liver disease (Lian and Fu, 2021).

Successful PPARγ target gene activation by detection of significantly increased adiponectin levels (Maeda et al., 2001; Iwaki et al., 2003) has previously been shown for our experimental setup as proof-of-principle (Katahira et al., 2021). This finding could be reproduced in the present approach as well. However, generally increased adiponectin levels in the course of CKD have been reported before, both in adenine-induced CKD rat models (Yu et al., 2014; Al et al., 2021) as well as in clinical studies (Martinez et al., 2013). In CKD patients, pioglitazone treatment led also to significantly increased plasma adiponectin levels together with decreased glucose levels (Zanchi et al., 2014) as we see in our model as well, indicative for successful PPARγ activation by pioglitazone. High adiponectin levels are considered as cardioprotective (Nesti et al., 2021). However, high levels of circulating adiponectin in patients with impaired kidney function have also been reported to be associated with adverse cardiovascular outcome, e.g., coronary artery events in the Korean population, whereas a causal relationship remains elusive (Suh et al., 2021).

Fetuin-A is a well-known negative acute phase protein. Reduced plasma and tissue fetuin-A levels are associated with tissue damage and dystrophic calcification (Rudloff et al., 2022). In this study however, plasma fetuin-A levels were similar at all time points and in all experimental groups. Importantly, the levels were never reduced below 50% of normal, the level present in hemizygous fetuin-A deficient mice, which do not spontaneously calcify (Schafer et al., 2003). Therefore, the fetuin-A plasma levels likely did not regulate calcification in the rats. Whether or not the protective effect of pioglitazone in CKD was fetuin-A mediated at earlier time points or locally in the damaged tissues requires further study.

### 4.2 Pioglitazone inhibits cardiovascular calcification associated with CKD and improves functional outcome

To acknowledge possible spatial differences in calcifying processes in our BVG, we analyzed wall segments and the aortic valvular part separately. Analysis of the aortic wall segments of BVG revealed CKD-induced calcifications rather in the media than in the intima region with an increase of calcification by time. Pioglitazone ameliorated the calcific burden notably at the 8 weeks time point, very similar to effects we have observed before in other models of accelerated degeneration of BVG (Assmann et al., 2021; Katahira et al., 2021; Assmann A. K. et al., 2022). Thus, pioglitazone can prevent from medial calcification in CKD which again might prevent further CVD or the progress of existing SVD. Besides these ameliorative effects of pioglitazone on BVG degeneration, CKD-induced intima hyperplasia (Chitalia et al., 2015; Hénaut et al., 2018) was also reduced by PPARγ activation. Attenuation of hyperplasia by pioglitazone has been shown before in clinical trials for drug-eluting stents (Marx et al., 2005; Hong et al., 2010) and for bioprosthetic grafts in own animal studies (Assmann et al., 2021; Assmann A. K. et al., 2022). Thus, pioglitazone administration in CKD patients on hemodialysis might also prevent from vascular access failure due to hyperplasia and calcification of hemodialysis access grafts (Vazquez-Padron and Allon, 2016).

Analysis of the valvular region of the grafts showed CKD-induced calcifications mainly in the leaflets and commissures. However, extenuating effects of pioglitazone were less obvious than in the aortic wall and rather apparent in the early time point indicating a later onset of degenerative effects in this part of the graft.

Protective effects of pioglitazone did not seem to be related with changes in the expression of chondro-osteogenic markers nor does adenine treatment alone led to increased expression except for OPN expression. Reports on increase of OPN, RUNX2, ALPL and PIT-1 expression in vascular tissue of CKD animals might attribute to additional high phosphorous diet (Tani et al., 2017). Moreover, unchanged BMP2, RUNX2 and PIT-1 gene expression in our grafts might also be due to unchanged RAGE levels (Li et al., 2012) or a RAGE-independent effect of pioglitazone (Belmokhtar et al., 2019).

Anti-inflammatory effects of pioglitazone in the context of CVD have been reported in clinical studies (Hong et al., 2010), *in vitro* studies (Ye et al., 2006; Sacks et al., 2011) and in animal models (Assmann et al., 2021; Wang et al., 2023). In clinical cohort studies, IL-6 presents as a crucial early mediator of inflammatory processes associated with major adverse cardiovascular events in CKD patients (Amdur et al., 2019; Batra et al., 2021). Thus, IL-6 is considered as a robust driving force for CVD in CKD patients, however, approaches using anti-inflammatory pharmacotherapy have not been convincing to date and clinical studies of targeting of IL-6 are still at the beginning (Huck et al., 2023). Systemic levels of circulating cytokines were beyond detection range, indicating a local action of PPARγ activation on IL-6 which has also recently been described for subdermal bioprosthesis material degeneration (Meng et al., 2021). In the herein presented model, CKD-induced early increase in IL-6 expression in BVG tissue could be attenuated by pioglitazone treatment. Since especially IL-6 seems to account for inflammatory induced graft degeneration (Sun et al., 2009; Ma et al., 2021; Meng et al., 2021), the observed effects of PPARγ activation in our model might be crucial for further studies to develop potential anti-degenerative therapies protecting CKD patients with bioprosthetic heart valve grafts from SVD.

*In vitro* and *in vivo* studies have shown that certain inflammatory target genes are modulated by pioglitazone in a PPARα-dependent manner (Orasanu et al., 2008). Since pioglitazone has been shown to be a weak PPARα agonist (Sakamoto et al., 2000) and characteristic PPARα-mediated effects like decreased triglyceride and LDL levels are missing in the pioglitazone-treated animals in our setting (Han and Qu, 2020; Krishnappa et al., 2020), potential impact of PPARα activation seems unlikely.

Pioglitazone treatment led to an improved functional outcome in terms of significant less AR in BVG of the pioglitazone treated group compared to control conditions as well as to BVG of CKD animals. Similar observations of this protective effect of pioglitazone treatment on BVG function and performance have been reported before (Assmann et al., 2021; Assmann A. K. et al., 2022).

### 4.3 Limitations of the study

Our *in vivo* study evaluates the impact of PPARγ activation by pioglitazone administration on BVG in an adenine-induced CKD model in rats. CKD, however, is a complex disease and our animal model does not entirely reflect the clinical setting present in patients with CKD in terms of co-morbidities such as diabetes or obesity as well as possible interference with additional medication besides PPARγ agonists. Therefore, further studies including these parameters are intended. Future experiments will also aim at large animal experiments for validation of our findings and to comprise anatomical factors as well as clinically relevant species for bioprosthetic graft engineering, e.g., porcine tissue, in a pre-clinical setting.

## 5 Conclusion

PPARγ activation by systemic pioglitazone administration ameliorates media and valvular calcification in BVG in a rat model of CKD and improves functional outcome. Potassium and phosphate levels are attenuated by pioglitazone treatment, possibly contributing to these anti-degenerative effects. Tissue expression of the early inflammatory mediator IL-6 is mitigated by pioglitazone, indicative for a possible cardio-renal protective mechanism of PPARγ activation. Further mechanistic research as well as pre-clinical studies in larger animal models are necessary to decipher the role of pioglitazone in bioprosthetic cardiovascular graft failure. Collectively, pioglitazone might be a valuable pharmaceutical tool for the prevention of SVD and thus bioprosthetic valve failure in CKD patients.

## Data Availability

The original contributions presented in the study are included in the article/Supplementary material, further inquiries can be directed to the corresponding author.
