# Supplementary material for: Pioglitazone treatment mitigates cardiovascular bioprosthetic degeneration in a chronic kidney disease model
Source: Front Pharmacol. 2024 Aug 8;15:1412169. doi: 10.3389/fphar.2024.1412169 (PMC11338925; doi:10.3389/fphar.2024.1412169)
Supplement: Supplementary file 1 [file DataSheet1.PDF]

Supplementary Material

Supplemental Figures

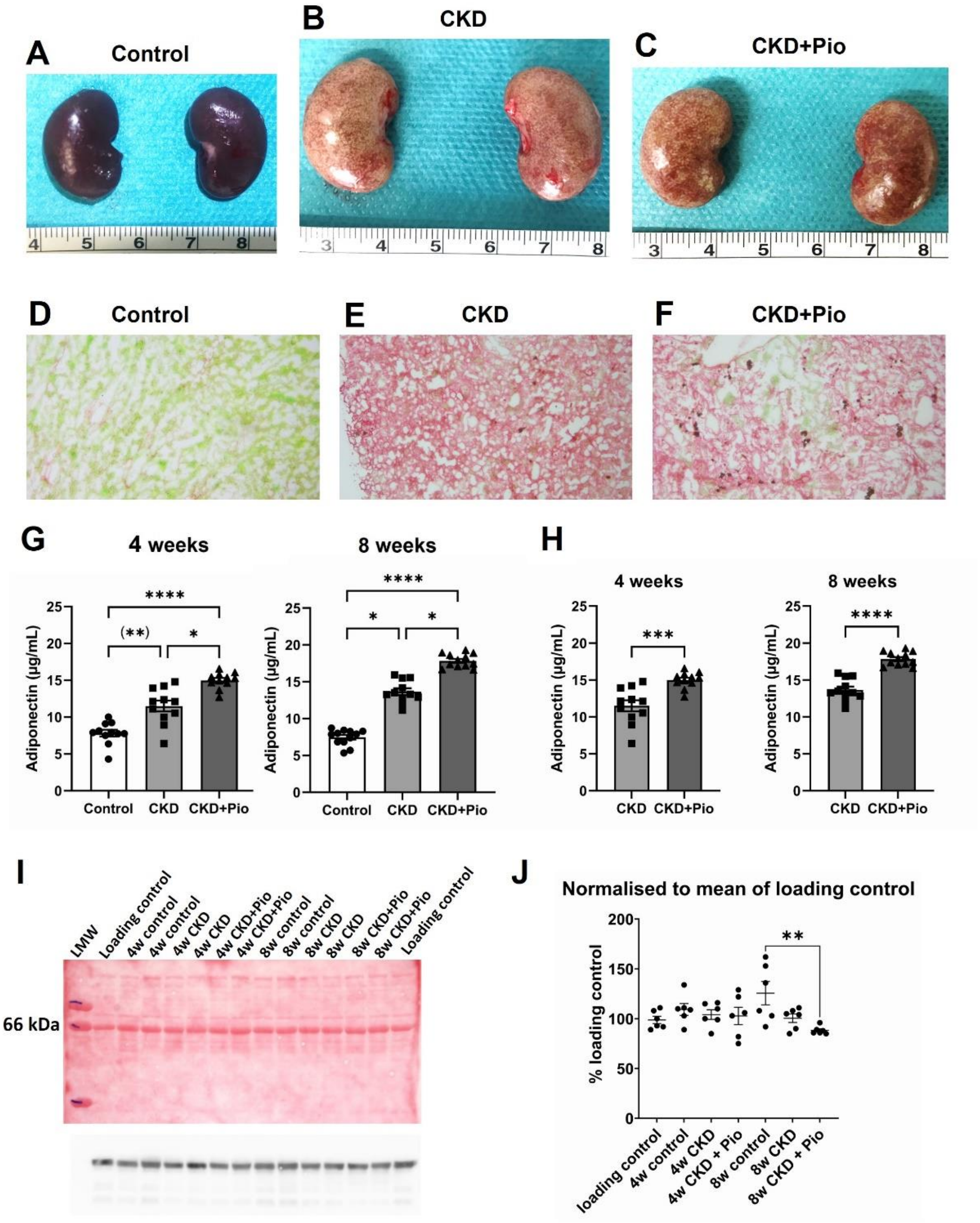

Supplemental Figure 1 Pathological changes in the kidneys, adiponectin and fetuin-A levels in plasma

(A-F) Pathological changes in the kidneys after CKD induction and treatment with Pio. Gross morphological inspection shows fibrotic structures and enlargement in the CKD group (B) compared to control (A) and an attenuated finding after Pio treatment (C). Picrosirius red/fast green staining reveals fibrotic changes by CKD treatment (E) compared to control (D) with a visually reduced amount in the Pio treated group (F). Adiponectin levels in plasma at explantation time point at 4 and 8 weeks (G) with values being mean ± standard error of mean. Adiponectin levels are significantly higher in CDK as well as in CKD+Pio groups at both time points, with significantly higher levels under pioglitazone treatment. (H) Direct comparison between CKD and CKD+Pio groups. Animals included: n=11-12 per subgroup. (I+J) SDS-PAGE and Western blot analysis of fetuin-A plasma levels. Representative images of ponceau S staining of the membrane (I; upper image) and detection of fetuin-A (I; lower image). Per gel, n=2 samples of each subgroup were used, resulting in a total of 3 gels. (J) ImageJ analysis of AU and analysis with GraphPad Prism show equal values of fetuin-A with differences between the control group and the CKD+Pio group at 8 weeks. CKD: chronic kidney disease; Pio: pioglitazone; *: $p<0.05$; **: $p<0.01$; ****: $p<0.0001$; (): pairwise comparison using Mann Whitney test.

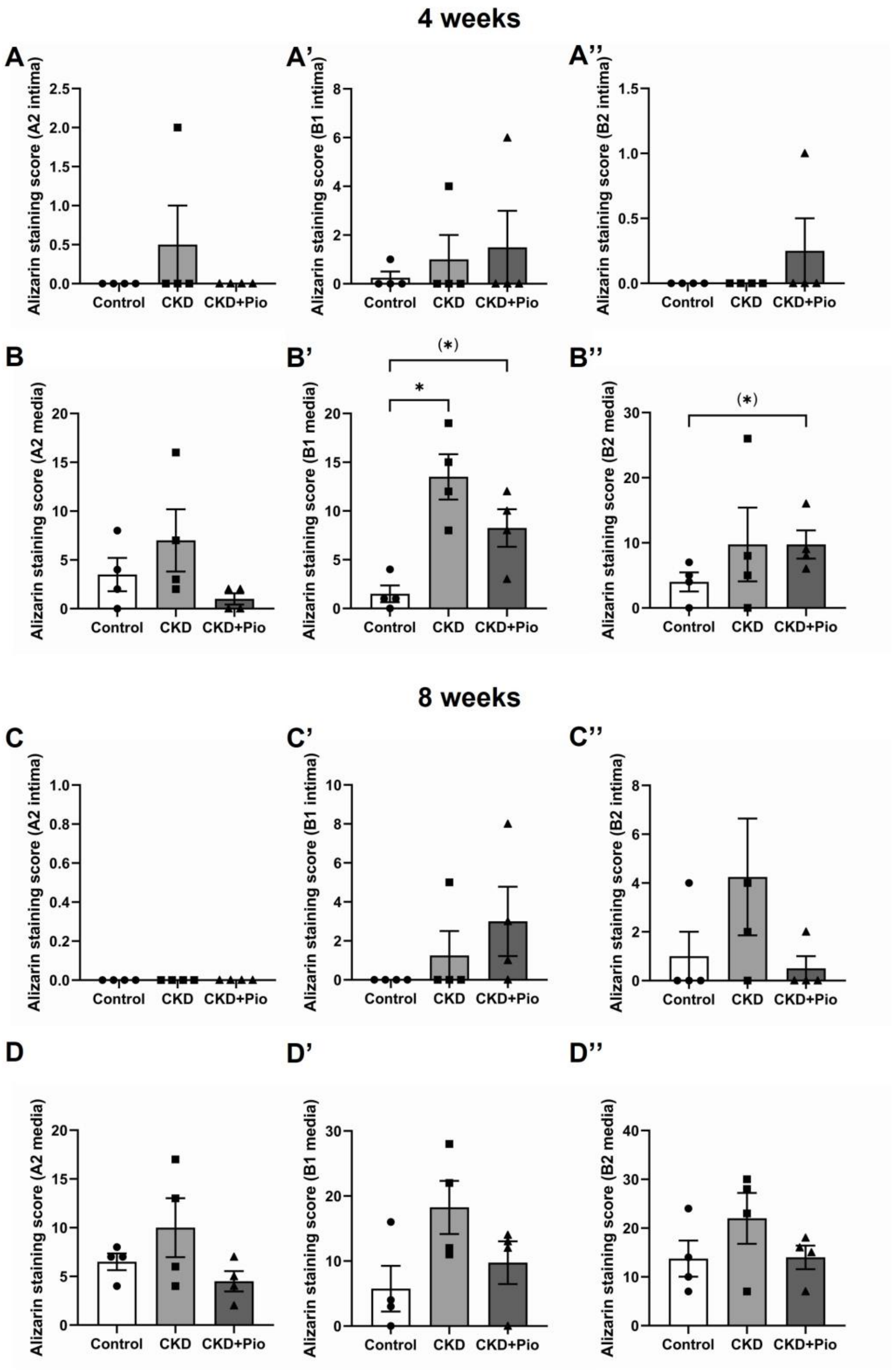

Supplemental Figure 2 Calcification in individual aortic wall regions by alizarin score

Quantification of calcification by alizarin staining score of regions A2-B2 of the grafts. (A-A’’) Intima calcification score of A2-B2 regions at 4 weeks. (B-B’’) Media calcification score of A2-B2 regions

at 4 weeks. (C-C’’) Intima calcification score of A2-B2 regions at 8 weeks. (D-D’’) Media calcification score of A2-B2 regions at 8 weeks. Animals included: n=4 in each subgroup with three sections of each sample. CKD: chronic kidney disease; Pio: pioglitazone; *: $p<0.05$; (): pairwise comparison using Mann Whitney test.

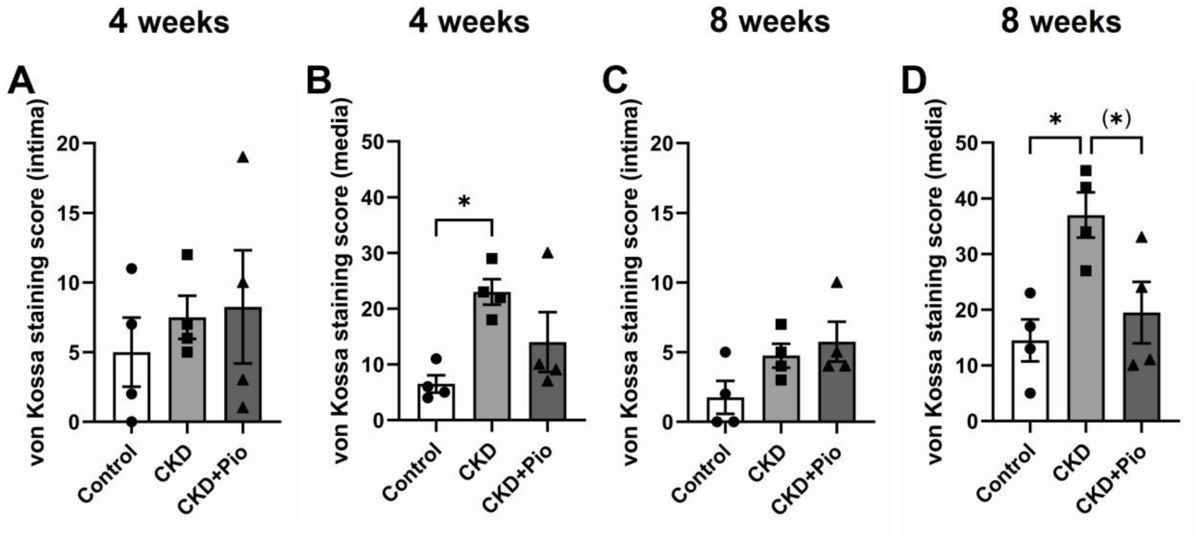

Supplemental Figure 3 Calcification of total aortic wall regions by von Kossa score

Quantification of calcification by von Kossa staining score of regions A2-B2 of the grafts in total. (A) Intima calcification score of total A2-B2 regions at 4 weeks. (B) Media calcification score of total A2-B2 regions at 4 weeks. (C) Intima calcification score of total A2-B2 regions at 8 weeks. (D) Media calcification score of total A2-B2 regions at 8 weeks. Animals included: n=4 in each subgroup with three sections of each sample. CKD: chronic kidney disease; Pio: pioglitazone; *: $p<0.05$; (): pairwise comparison using Mann Whitney test.

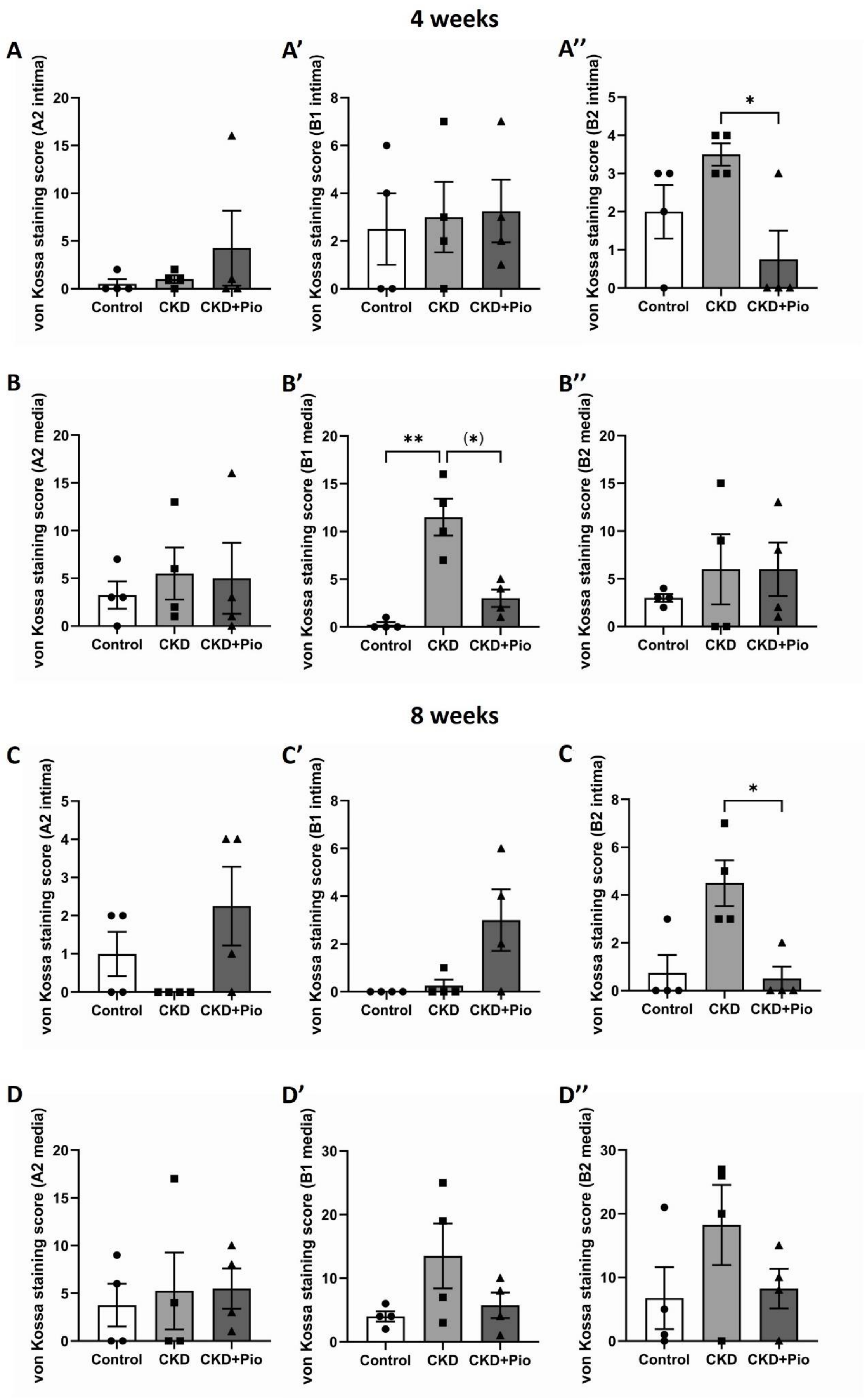
4 weeks
A
von Kossa staining score (A2 intima)
A’
von Kossa staining score (B1 intima)
A’’
von Kossa staining score (B2 intima)
*
B
von Kossa staining score (A2 media)
B’
von Kossa staining score (B1 media)
**
(*)
B’’
von Kossa staining score (B2 media)
8 weeks
C
von Kossa staining score (A2 intima)
C’
von Kossa staining score (B1 intima)
C
von Kossa staining score (B2 intima)
*
D
von Kossa staining score (A2 media)
D’
von Kossa staining score (B1 media)
D’’
von Kossa staining score (B2 media)
Control
CKD
CKD+Pio

Supplemental Figure 4 Calcification in individual aortic wall regions by von Kossa score

Quantification of calcification by von Kossa staining score of regions A2-B2 of the grafts. (A-A’’) Intima calcification score of A2-B2 regions at 4 weeks. (B-B’’) Media calcification score of A2-B2 regions at 4 weeks. (C-C’’) Intima calcification score of A2-B2 regions at 8 weeks. (D-D’’) Media calcification score of A2-B2 regions at 8 weeks. Animals included: n=4 in each subgroup with three sections of each sample. CKD: chronic kidney disease; Pio: pioglitazone; *: $p<0.05$; **: $p<0.01$; (): pairwise comparison using Mann Whitney test.

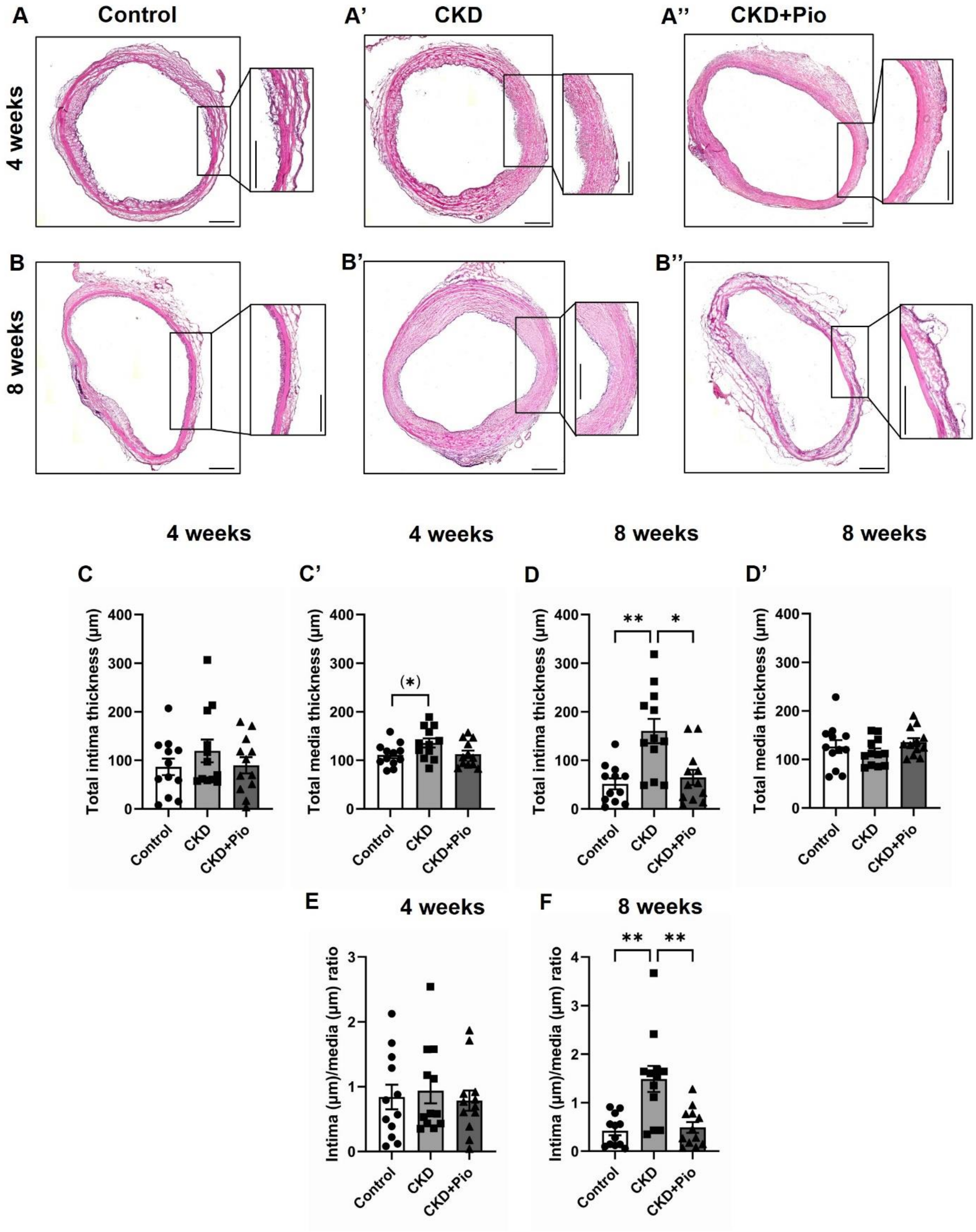

Supplemental Figure 5 Intima hyperplasia

HE staining (A-B'') and quantification of total intima and media thickness of the grafts (C-F). After 4 weeks, intima and media thickness are unchanged. After 8 weeks, intima hyperplasia in the CKD group

is mitigated by pioglitazone, while media thickness stays unchanged. Intima/media ratio is decreased by pioglitazone after 8 weeks. Animals included: n=4 in each subgroup with three sections of each sample. CKD: chronic kidney disease; Pio: pioglitazone; *: $p<0.05$; **: $p<0.01$; (): pairwise comparison using Mann Whitney test. Bars: 400 µm.

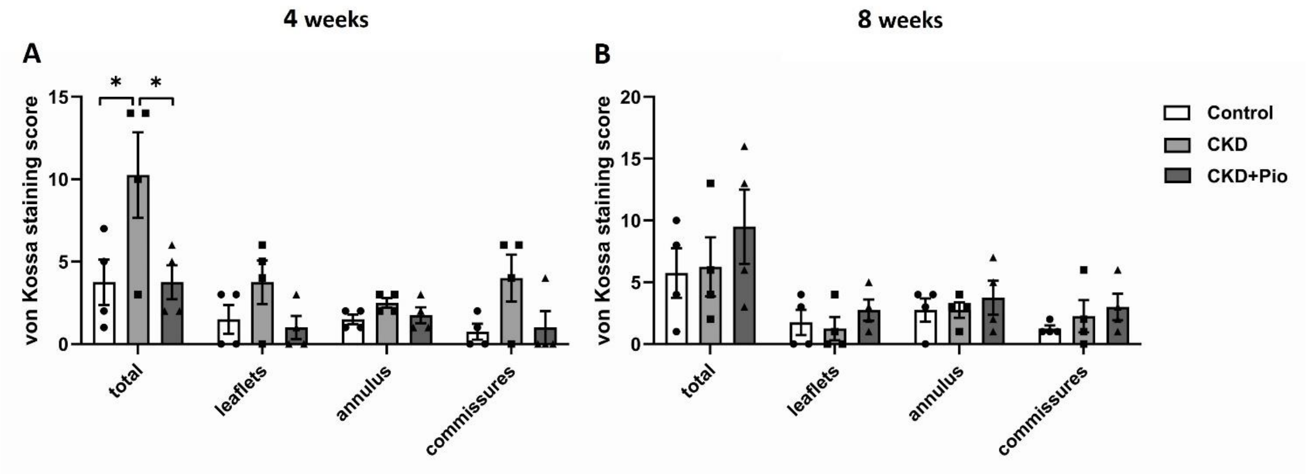

Supplemental Figure 6 Calcification in individual aortic valve regions by von Kossa score

Quantification of calcification by von Kossa staining score of A1 valvular region of the grafts. (A) Calcification score of valvular regions and in total at 4 weeks. (B) Calcification score of valvular regions and in total at 8 weeks. Animals included: n=4 in each subgroup with three sections of each sample. CKD: chronic kidney disease; Pio: pioglitazone; *: p<0.05.

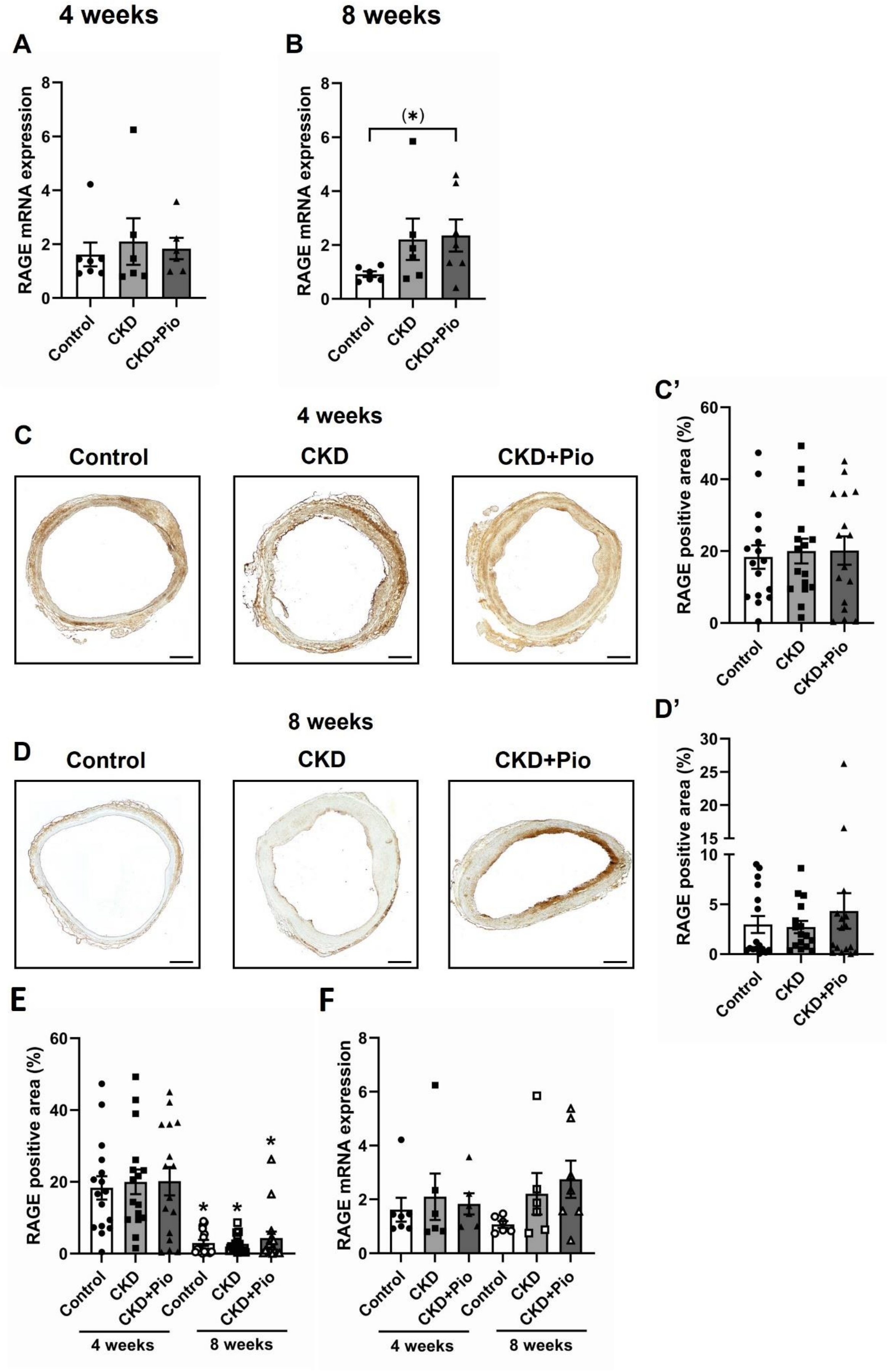

4 weeks
8 weeks
A
B
RAGE mRNA expression
(*)
Control
CKD
CKD+Pio
C
4 weeks
Control
CKD
CKD+Pio
C'
RAGE positive area (%)
D
8 weeks
Control
CKD
CKD+Pio
D'
RAGE positive area (%)
E
RAGE positive area (%)
4 weeks
8 weeks
F
RAGE mRNA expression
4 weeks
8 weeks

Supplemental Figure 7 RAGE gene and protein expression

Gene expression analysis of RAGE at explantation time point at 4 and 8 weeks with values being mean ± standard error of mean. RAGE expression is altered at 8 weeks. RAGE immunohistology (C+D) and quantification of staining (C'+D'). (E) RAGE positive areas in % of stained sections of total valve-bearing bioprosthetic conduit grafts at 4 and 8 weeks after implantation. (F) RAGE gene expression of total valve-bearing bioprosthetic conduit grafts at 4 and 8 weeks after implantation. Animals included for mRNA expression analysis: n=6-7 in each subgroup. Animals included for immunohistology analysis: n=4 in each subgroup with three sections of each sample. CKD: chronic kidney disease; Pio: pioglitazone; *: $p<0.05$; (): pairwise comparison using Mann Whitney test.
